# Effects of Diminished NADPH:cytochrome P450 Reductase in Human Hepatocytes on Lipid and Bile Acid Homeostasis

**DOI:** 10.3389/fphar.2021.769703

**Published:** 2021-11-15

**Authors:** Tamara Heintze, Denise Wilhelm, Thierry Schmidlin, Ute Hofmann, Ulrich M. Zanger, Matthias Schwab, Kathrin Klein

**Affiliations:** ^1^ Margarete Fischer-Bosch Institute of Clinical Pharmacology, Stuttgart, Germany; ^2^ Eberhard Karls University, Tübingen, Germany; ^3^ Nuffield Department of Medicine, University of Oxford, Oxford, United Kingdom; ^4^ Departments of Clinical Pharmacology and Biochemistry and Pharmacy, University of Tuebingen, Tübingen, Germany; ^5^ Cluster of Excellence IFIT (EXC 2180) “Image-Guided and Functionally Instructed Tumor Therapies”, University of Tübingen, Tübingen, Germany

**Keywords:** HepaRG, CRISPR/Cas9, NADPH cytochrome P450 reductase, bile acid metabolism, cholesterol biosynthesis, proteomics

## Abstract

NADPH:cytochrome P450 oxidoreductase (POR) is the obligate electron donor for microsomal cytochrome P450 (CYP) enzymes involved in the biosynthesis of endogenous substances like bile acids and other steroids as well as in the oxidative metabolism of xenobiotics. P450 oxidoreductase also supports other redox enzymes in fatty acid and cholesterol pathways. Recently, we have established CRISPR/Cas9-mediated POR knockdown in a human hepatic cell model, HepaRG, and demonstrated the differential effects of limited POR expression on CYP activity. The aim of the present work was to systematically investigate the impact of POR knockdown with a focus on the expression of ADME (absorption, distribution, metabolism, and excretion) genes and related regulators. Functional consequences have been assessed using quantitative mass spectrometry for targeted metabolomics covering bile acids, and cholesterol and its precursors, and for untargeted proteomics. In addition to the previously described alteration of RNA expression of CYP genes, we showed significant downregulation of transcriptional regulators of drug metabolism and transport, including NR1I3 (CAR), NR1I2 (PXR), NR1H4 (FXR), and NR1H3 (LXRα) in cells with *POR* gene disruption. Furthermore, POR knockdown resulted in deregulated bile acid and cholesterol biosynthesis demonstrated by low levels of cholic acid derivates and increased concentrations of chenodeoxycholic acid derivates, respectively. Systemic effects of POR knockdown on global protein expression were indicated by downregulation of several metabolic pathways including lipid metabolism and biological oxidation reactions. The deduced protein network map corroborates CYP enzymes as direct interaction partners, whereas changes in lipid metabolism and homeostasis are the result of indirect effects. In summary, our results emphasize a widespread role of POR in various metabolic pathways and provide the first human data on the effects of diminished POR expression on drug and endogenous metabolism in a genomeedited HepaRG cell model.

## Introduction

NADPH:Cytochrome P450 reductase (POR) is a ubiquitous microsomal electron transport protein essential for cytochrome P450 (CYP) mediated oxidative metabolism of xenobiotics and physiological processes. This includes biosynthesis of numerous endogenous compounds especially steroids such as cholesterol and bile acids (Riddick et al., 2013). POR plays also a pivotal role for other important redox partners like heme oxygenase HMOX1, squalene monooxygenase or cytochrome b5 CYB5A (Porter, 2012; Pandey and Flück, 2013). Genomic deletion of *Por* in mice leads to embryonal death around day 13 due to severe disturbances in retinoid and cholesterol homeostasis (Shen et al., 2002; Otto et al., 2003; Schmidt et al., 2009). So far effects of *Por* knockout in liver on drug as well as endogenous metabolism were extensively studied in mouse models (Gu et al., 2003; Henderson et al., 2003; Wang et al., 2005; Weng et al., 2005; Finn et al., 2009; Cheng et al., 2014b). In addition to diminished CYP activity levels (Gu et al., 2003; Henderson et al., 2003; Finn et al., 2007), these mice developed severe hepatic lipidosis as well as reduced circulating cholesterol, triglyceride levels and gallbladder bile volume (Gu et al., 2003; Henderson et al., 2003; Wu et al., 2003). Moreover, in-depth studies in liver specific *Por* knockout mice revealed diverse effects on bile acid synthesis. While total amounts of bile acids in mouse liver were only slightly decreased, concentrations of chenodeoxycholic acid (CDCA) and its gut microbial product lithocholic acid (LCA) were increased, probably due to induction of the alternative bile acid synthesis pathway (Cheng et al., 2014b; Gethings et al., 2021). In humans the multistep bile acid biosynthesis is mainly mediated by CYPs 7A1, 7B1, 8B1 and the mitochondrial, POR-independent CYP27A1, resulting in a substantially different composition of the bile acid pool. In contrast mice are displaying a more hydrophilic complement of bile acid species (Li and Dawson, 2019).

Bile acids act as endogenous ligands for FXR, PXR and G proteins-coupled BA receptor (TGR5 (Gpbar1)), which regulate homeostasis of xenobiotics and lipid, glucose and energy metabolism (Pols et al., 2011; Chiang and Ferrell, 2018). Gene expression analyses of liver specific *Por* knockout mice generally indicate an induction of drug metabolizing as well as bile acid synthesizing *Cyp* expression (Wang et al., 2005; Weng et al., 2005; Mutch et al., 2007; Henderson et al., 2013). This effect is ascribed to changed bile acid signaling arising from altered bile acid composition (Wang et al., 2005; Weng et al., 2005; Mutch et al., 2007; Henderson et al., 2013). Increased hepatic lipid accumulation as a result of impaired lipid secretion coupled with increased lipid uptake has also been attributed to changed bile acid signaling (Weng et al., 2005; Mutch et al., 2007).

In humans certain POR gene mutations lead to deficient POR (PORD) characterized by disordered steroidogenesis with broad phenotypic spectrum including cortisol deficiency, altered sex steroid synthesis, disorders of sex development and skeletal malformations resembling the Antley-Bixler syndrome phenotype (Flück and Pandey, 2011; Fukami and Ogata, 2014; Miller, 2018). Medical treatment of PORD patients consisting of glucocorticoid and sex steroid replacement therapy requires individually tailored dosing due to diminished CYP mediated hepatic drug metabolism (Tomalik-Scharte et al., 2010; Miller et al., 2011). Yet, no phenotypic changes of livers in PORD patients comparable to the lipidosis observed in mouse liver were reported so far (Miller, 2018). Furthermore, translation of findings from mouse models to the human system is limited due to differences in the composition of CYP enzymes (Takahashi et al., 2016) or the bile acid pool (Li and Dawson, 2019; Straniero et al., 2020). For a better understanding of the role of POR in human liver metabolism, meaningful human cell models are needed.

To study systematically the effect of diminished POR levels on drug metabolising CYP activity and expression in a human cell background, we recently established two genetic *POR* HepaRG knockout cell lines using CRISPR/Cas9 technology (Heintze et al., 2021). HepaRG cells have the unique ability to differentiate into hepatocyte- and biliary-like cells (Gripon et al., 2002). Compared to other hepatoma cell lines, HepaRG cells demonstrate stable and functional expression of a broad range of liver specific genes comparable to primary human hepatocytes (Rogue et al., 2012). These include several CYP enzymes as well as phase II enzymes, drug transporters, and liver-specific transcription factors comprising dedicated ligand-activated nuclear receptors. HepaRG cells have therefore become widely accepted as a highly useful model to study various aspects of drug metabolism, transport and its regulation (Kanebratt and Andersson, 2008; Andersson et al., 2012; Klein et al., 2015; Rubin et al., 2015; Tolosa et al., 2016; Tanner et al., 2018; Kugler et al., 2020). Best to mimic the human situation, HepaRG cells are ideally suited to investigate the effects of diminished POR on drug as well as endogenous metabolism. We recently showed differential effects on CYP activity levels and isoform dependent effects on CYP expression in the newly established genetic *POR* knockout HepaRG cell model (Heintze et al., 2021). Most strikingly, contrasting to increased CYP expression seen in mice, CYP expression levels were generally decreased except for CYPs 1A1 and 1A2.

The aim of our present study was to analyze comprehensively the impact of diminished POR levels in the HepaRG cell background with focus on genes involved in the absorption, distribution, metabolism, and excretion (ADME) and related regulators. We analysed mRNA expression, measured intracellular cholesterol precursers and bile acid secretion by mass spectrometry and protein expression using an untargeted proteomics approach. Our genetic *POR* knockout HepaRG cell model provides first human data on changes in gene and protein expression and the metabolite composition, which reveal marked differences to rodents. The results indicate widespread effects on gene transcription and protein abundance in certain pathways that may reflect coordinated responses to changed endogenous metabolites like bile acids.

## Materials and Methods

### HepaRG Cell Culture

HepaRG cells (batch HPR101007, passage no. 12) (Gripon et al., 2002) were obtained from Biopredic International (Rennes, France) and cultured as described (Klein et al., 2015; Heintze et al., 2021).

### CRISPR/Cas9 Genome Editing

Generation and propagation of genetic *POR* knockout HepaRG cell lines HepaRG^−POR#1^, HepaRG^−POR#2^ and HepaRG^VC^ was previously described (Heintze et al., 2021). In short, a lentiviral CRISPR/Cas9 system was used for high transduction efficiency of two designed guide RNAs (sgRNA) POR#1 and POR#2 targeting POR gene with subsequent puromycin selection. Genome editing was confirmed by T7 endonuclease one digest. For downstream analysis of activity, mRNA expression and protein composition cells were differentiated by DMSO treatment for 2 weeks as described (Heintze et al., 2021).

### Quantitative Real-Time PCR for Gene Expression Analysis

100–200 ng of total RNA (isolated using RNeasy Plus Kit (Qiagen, Hilden, Germany) was reverse transcribed with TaqMan Reverse Transcription Reagents (Applied Biosystems, Foster City, United States). cDNA was preamplified using TaqMan PreAmp Mastermix (Applied Biosystems) and expression of 82 selected genes was quantified using the Biomark HD system (Fluidigm, San Francisco, United States) with a 48:48 Dynamic Array Chip (Fluidigm). Expression levels were determined from six independent cell expansions for each cell line and normalized to the geometric mean of glyceraldehyde-3-phosphate dehydrogenase (GAPDH), RPLP0 and β-actin (ACTB) expression according to Vandesompele et al. (2002) and relatively quantified using the ΔΔct method.

### Quantification of Bile Acids

Bile acid secretion into medium was measured after incubation of differentiated cells with serum free medium for 24 and 48 h. Secreted cholic acids (GCA, TCA) as well as chenodeoxycholic acid derivates (GCDCA, TCDCA) were quantified in the medium by negative electrospray (ESI) liquid chromatography tandem mass spectrometry (LC-MS/MS) in multiple-reaction-monitoring (MRM) mode on an Agilent 6460 triple quadrupole mass spectrometer (Agilent Technologies, Waldbronn, Germany) coupled to an Agilent 1290 HPLC system as described elsewhere (Ghallab et al., 2019).

### Quantification of Cholesterol and Its Precursors

Cholesterol, lathosterol and lanosterol were quantified by GC-MS analysis on a 5975C inert XL MSD, coupled to a 7890A GC (Agilent) as described previously (Maier et al., 2009), with minor modifications. Briefly, the cell pellet was spiked with internal standards [^2^H_5_]cholesterol and [^2^H_7_]lathosterol, extracted with hexane:2-propanol 3:2 v/v, and saponified with 1 M NaOH in 90% ethanol followed by a second extraction step with cyclohexane. The organic phase was evaporated and the *tert*-butyldimethylsilyl derivatives prepared by addition of 20 µL of N-*tert*-butyldimethylsilyl-N-methyltrifluoroacetamide (MBDSTFA) and 20 µL of DMF. Cholesterol and lathosterol were measured in selected ion monitoring (SIM) mode at m/z 443.4, lanosterol at m/z 393.4 [^2^H_5_]cholesterol at m/z 448.4, and [^2^H_7_]lathosterol at m/z 450.4. Calibration samples prepared directly from the working solutions, were worked up as described above, and analyzed together with the unknown samples. Calibration curves based on internal standard calibration were obtained by weighted (1/x) linear regression for the peak-area ratio of the analyte to the respective internal standard against the amount of the analyte. The concentration of the analytes in unknown samples was obtained from the regression line.

### Nile Red Assay

Lipid droplet quantification by Nile Red staining was performed after incubation of cells with serum free medium for 5 days. Nile Red (15 μg/μL) (Santa Cruz Biotechnology, Dallas, United States) was added to the cell medium for 20 min at 37°C. Nile Red fluorescence was analyzed at 580 nm using an EnSpire Mulitmode plate reader (PerkinElmer, Waltham, United States). For normalization cell vitality was assessed using the alamarBlue assay (ThermoFisher, Waltham, United States) according to the manufacturer’s instructions.

### Proteomics

#### Experimental Setup and Sample Preparation

Three biological replicates for each condition were used and each sample was analyzed twice with LC-MS/MS making a total of six replicates per condition. Cells were seeded in 10 cm dishes, grown to full confluency, and differentiated according to the protocol described above. Cells were washed twice with PBS, scraped off and pelleted. Cell pellets (6 – 8 x 10^6^ cells) were lysed in 50 µL lysis buffer (LB, 8 M urea in 50 mM ammonium bicarbonate (ABC), supplemented with 1x cOmplete Mini™ protease inhibitor (Sigma Aldrich, Schnelldorf, Germany)) by sonication for 15 min at high level (BIORUPTOR^®^ UCD-200TM-EX, Diagenode, Belgium) and centrifuged for 15 min at 20,000 x *g* at 4°C. Protein concentration was determined by Bradford protein assay (Bio-Rad Laboratories, Hercules, United States) measured at 595 nm (EnSpire™ Multimode Plate Reader, Perkin Elmer). Optimal protein amounts for subsequent sample workups were previously determined to be in the range of 200 µg–1 mg, here samples containing 300 µg of protein were diluted with LB to a final protein concentration of 3 μg/μL. Cystein reduction and alkylation was performed by incubating with 5 mM dithiotreitol (DTT) at 60 °C for 45 min at 300 rpm (ThermoMixer C (Eppendorf AG, Hamburg, Germany)) followed by incubation with 20 mM iodoacetamide (IAA) in the dark for 45 min. Excess IAA was quenched with 2.5 µL of 200 mM DTT for 30 min in the dark. Urea concentration was lowered to 1 M with ABC and the samples digested with sequence-grade modified trypsin (Promega, Madison, United States) at an enzyme to protein ratio of 1:50 for 14–18 h at 37 °C. Digestion was quenched by acidifying to 4% formic acid (FA) (v/v). Samples were desalted by Sep-Pak c18, 1cc cartridges (Waters, Eschborn, Germany), dried down *in vacuo* (RVC 2–25 CD plus, Christ Martin GmbH, Osterode, Germany) and stored at −80°C.

#### LC-MS/MS Analysis

Methods for LC-MS/MS analysis were previously optimized for injection amounts of 30–100 µg peptides, here samples were reconstituted in 90 µL of 0.1% FA in water of which 15 µL were used per LC-MS injection, loading a total amount of 45 µg peptides on column. Proteomics analysis was performed using a 1290 infinity HPLC system (Agilent Technologies) coupled to a 6550 iFunnel QTOF (Agilent Technologies). Peptides were separated on an AdvanceBio Peptide Mapping column (120°A, 2.1 × 250 mm, 2.7 µm, Agilent Technologies) at a flow of 0.4 ml/min using 0.1% FA in water as solvent A and 0.1% FA in acetonitrile as solvent B. Peptides were separated for 60 min on a gradient from 3 to 40% B followed by ramping up to 65% B in 3 min. The column was washed at 100% B for 5 min followed by re-equilibration. Column oven temperature was set at 50°C. Mass spectrometer methods were run in data-dependent acquisition mode. All scans were acquired at high resolution setting. Survey scans were acquired from 375–1,250 m/z at a scan rate of eight spectra/s and fragmentation was induced for the top 20 peaks per cycle using a 6 s dynamic exclusion. Further filter criteria included a charge state of 2 + or 3+ and a minimum intensity threshold of 1 x 10^3^. Ions selected for fragmentation were isolated in a narrow isolation width of ∼1.3 amu and subjected to CID fragmentation using ramped collision energy. MS/MS scans were acquired from 100–1,500 m/z at an abundance based scan speed aiming for a target of 1 x 10^5^ counts per spectrum with a maximum scan rate of 10 spectra/s (Chu et al., 2017). The mass spectrometry proteomics data have been deposited in the ProteomeXchange Consortium via the PRIDE partner repository (http://www.ebi.ac.uk/pride; accession number PXD028577).

#### Proteomics Data Processing and Analysis

Protein identification and label-free quantification was performed by PEAKS 10.5 software (Bioinformatics Solutions Inc.). PEAKS *de novo* assisted sequencing was implemented prior to the database search. Search parameters for the database searches include precursor ion matching using a mass tolerance of 10 ppm and a fragment ion tolerance of 0.03 Da. Databases for the human proteome sequences were retrieved from uniprot.com (version January 2019). Peptide specificity was set to tryptic digest allowing for ≤2 missed cleavages, using carbamidomethylation as fixed modification and methionine oxidation as variable modification. PTM searches and SPIDER searches were allowed for the detection of additional posttranslational modifications and single amino acid exchanges. A false discovery rate of 1% was set using a parallel decoy database search. Peptides were quantified by the PEAKS Quant module using the top three peptides per protein exceeding a peptide quality threshold of eight for protein quantification. Relative abundances of each protein were calculated by normalization to the average of all samples. Quantitative values for proteins were subsequently used for correlation and PCA analysis, restricted to lists of proteins successfully quantified in the respective pair of LC-MS runs for correlation plots or in all 18 LC-MS runs for PCA. Analysis and visualization of correlation and PCA was performed in R.

### Statistical Analyses and Graphic Tools

Volcano plots and statistical analyses between HepaRG^−POR^ cells and HepaRG^VC^ were performed using Graphpad Prism V9 software or Analyst Module V13 (Genedata, Basel, Switzerland). Measure duplicates of proteome data were averaged and normalized to top three peptides and two groups comparison *t*-test and Benjamini-Hochberg adjustment was calculated. The significance level was set to *p* < 0.05, and effect size threshold was set to ≥0.6 (corresponding to fold change of about 2). Results are shown as means ± standard deviation (SD). We used Reactome (reactome.org), a curated database of pathways and reactions in human biology (pathway browser version 3.7, Reactome database release 77, from June 29th, 2021) for quantitative pathway analysis. Gene set analysis through limma R package algorithm Camera (reactome.org (Griss et al., 2020)) was performed on proteomics data with arithmetic means of two determinations for each sample of the three sets of HepaRG^VC^, HepaRG^−POR#1^ and HepaRG^−POR#2^. From 2,075 quantified proteins occurring in all cell lines, 1,820 entities were recognized and mapped to pathways including disease pathways. Expression differences of overrepresented pathways from both types of HepaRG^−POR^ cells compared to HepaRG^VC^ cells were illustrated as Voronoi graph in reactome.org. Protein-protein interaction networks were evaluated using STRING (https://string-db.org/, Szklarczyk et al., 2019).

## Results

### Effects of POR Knockdown on RNA Expression

**FIGURE 1:**
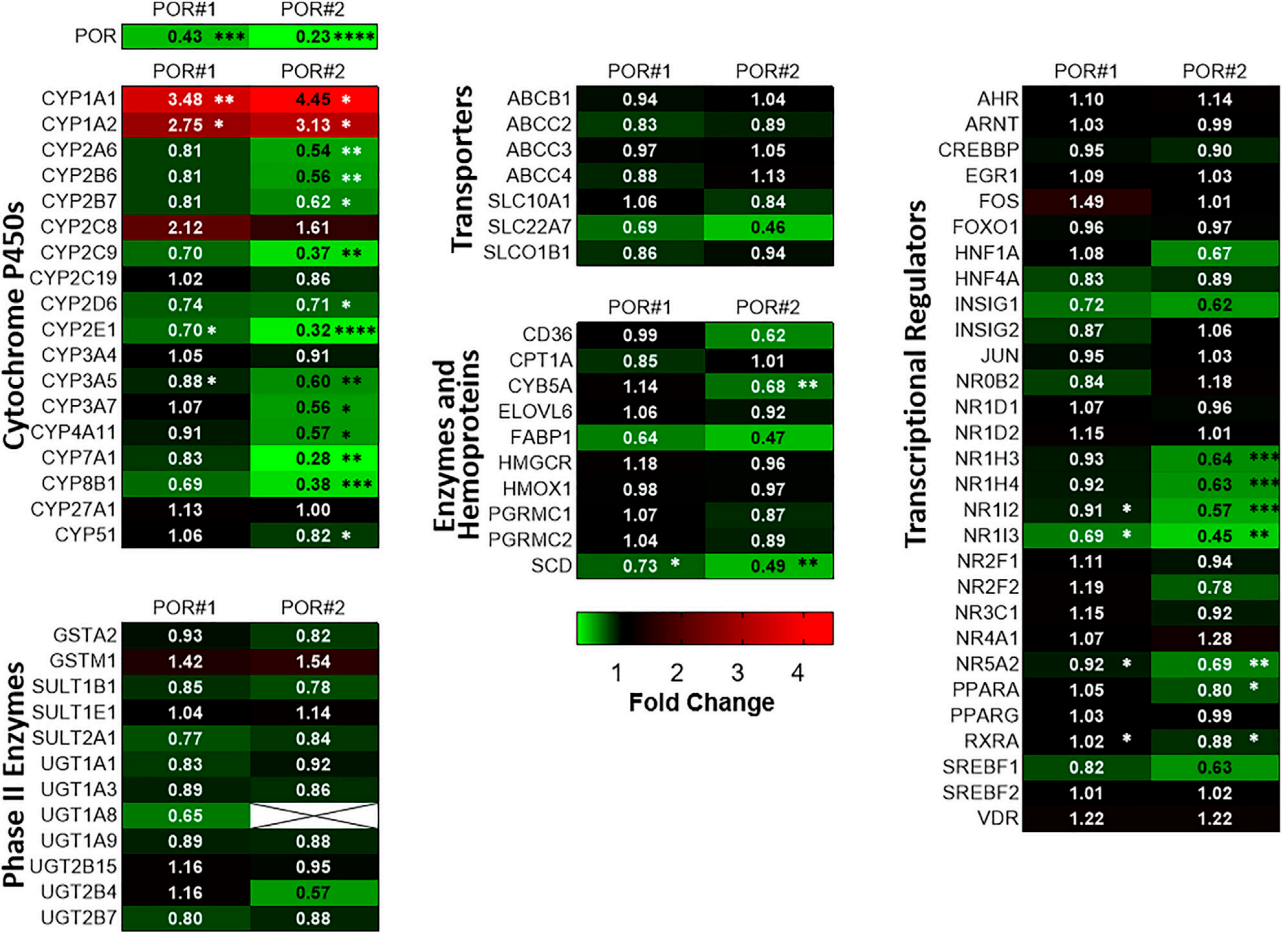
Differential gene expression profiles in HepRG^−POR^ cells. Gene expression analysis of HepaRG cells transduced with sgRNAs POR#1 and POR#2 and vector control (VC) and differentiated for 2 weeks was performed by qPCR. Data of six independent experiments were normalized to the geometric mean of GAPDH, RPLP0 and ACTB expression. The expression of the indicated genes of different classes as indicated is shown as fold changes relative to VC and represented as heat map (color code indicated below). Statistical significance was assessed by paired *t*-test (*p < 0.05, **p < 0.01, ***p < 0.001. ****p < 0.0001).

Figure 1 shows mRNA profiles of both types of HepaRG^−POR^ cells in comparison to HepaRG^VC^. As previously described, the most prominent effect was observed for POR expression (see Supplementary Figure S1 taken from Heintze et al., 2021). In addition to previously analyzed drug metabolizing CYP enzymes (Heintze et al., 2021), other drug metabolizing as well as endogenous substrate metabolizing CYPs also showed diverse effects. For CYPs 2A6, 2B7, 2E1, 3A5 and 3A7 a decrease in mRNA expression was observed with generally stronger effects seen in HepaRG^−POR#2^ (Figure 1, upper left panel). This further emphasized the uniqueness of increased mRNA expression among the phase I enzymes as only observed for CYP1A1 and CYP1A2 (3.5-fold and 4.5-fold, respectively; (Heintze et al., 2021)). In addition to CYPs 2E1 and 2C9, expression of two CYP isoforms involved in bile acid synthesis, CYP7A1 and 8B1, were among the four strongest decreased phase I enzymes with up to 0.28-fold lower expression levels. mRNA expression of two other CYPs 4A11 and 51 involved in fatty acid metabolism and cholesterol biosynthesis, respectively, was also decreased, albeit CYP51 was only marginally affected in HepaRG^−POR#2^ cells. In contrast, mRNA expression of CYP27A1, which catalyzes the first step of the alternative bile acid synthesis pathway, was not affected by POR knockout. (Figure 1, upper left panel).

We measured in parallel the expression levels of a broad spectrum of other potentially affected ADME genes in both types of HepaRG^−POR^ cells compared to HepaRG^VC^ cells. Generally, gene expression was changed in the same direction and none of the genes was upregulated. Again, in HepaRG^−POR#2^ cells slightly stronger effects were observed. Phase II enzymes, as well as transporters were generally less strongly affected by diminished POR compared to CYP genes and no significant effects were observed. In addition to CYP4A11 we observed significant downregulation of two genes involved in fatty acid processing, namely stearoyl-CoA desaturase (SCD) and cytochrome b5 (CYB5A), a required cofactor for several fatty acid desaturase reactions. Expression of the fatty acid binding protein (FABP1) was also strongly reduced, but this difference was not statistically significant (Figure 1, lower left and middle panels).

Interestingly, we noticed several consistent patterns of expression changes for transcriptional regulators. Thus, several regulators of hepatic gene expression were consistently downregulated up to 0.45-fold in both HepaRG^−POR^ cell lines, including NR1I3 (CAR), NR1H4 (FXR), NR1H3 (LXRα), PPARA (PPARα), NR1I2 (PXR), RXRA (RXRα). NR5A2 (LRH-1) as regulator involved in sterol signaling was also downregulated significantly (Figure 1, panel on the right). Sterol Regulatory Element Binding Transcription Factor 1 (SREBF1), an important transcription factor involved in lipid homeostasis and sterol biosynthesis had decreased mRNA expression, but this effect was statistically not significant. By contrast, expression of AHR/ARNT as transcriptional components of the Ah receptor pathway regulating CYP1A1/1A2 expression, was not changed significantly.

### Effects of POR Knockdown on Sterol and Bile Acid Synthesis

**FIGURE 2:**
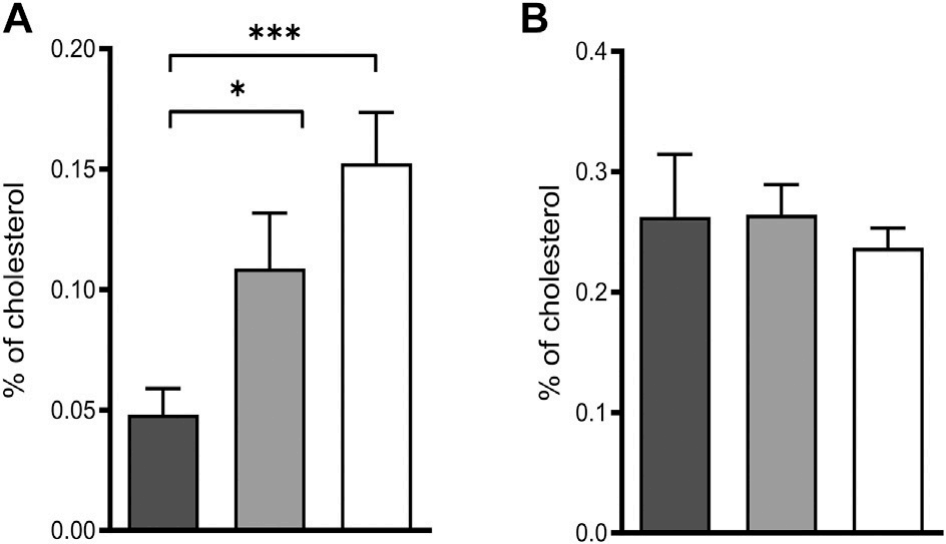
Intermediates of cholesterol biosynthesis in HepaRG cells. Relative lanosterol **(A)** and lathosterol **(B)** concentrations as a percentage of cholesterol in HepaRG cells transduced with vector control (black) or sgRNAs POR#1 (light grey) and POR#2 (white) and differentiated for 2 weeks were analyzed by GC-MS. Shown are means and SD of four independent measurements. Statistical significance was assessed by one-way ANOVA with Bonferroni correction (*p < 0.05, **p < 0.01, ***p < 0.001).

Deficiency of POR leads to blockage of the sterol synthesis pathway at lanosterol demethylase (CYP51) resulting in the accumulation of lanosterol in hepatocytes of hepatic POR-null mice or in rat hepatoma cells with suppression of POR expression by siRNA (Porter et al., 2011). In fact, we could reproduce the accumulation of lanosterol (Figure 2A) in POR knockdown HepaRG cells and found unchanged levels of the cholesterol precursor lathosterol (Figure 2B).

**FIGURE 3:**
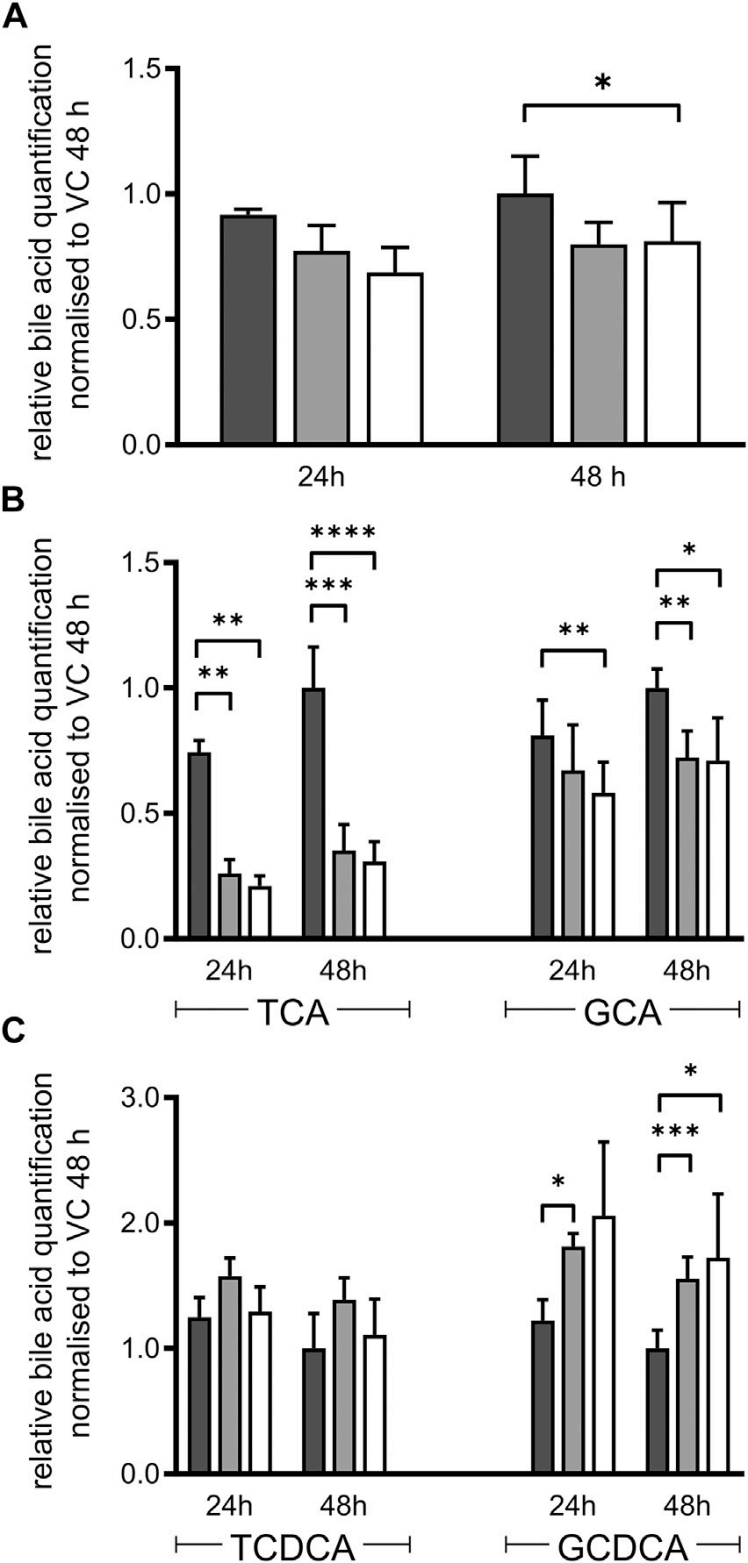
Bile acid secretion of HepaRG cells. Relative bile acid secretion into medium of HepaRG cells transduced with vector control (black) or sgRNAs POR#1 (light grey) and POR#2 (white) and differentiated for 2 weeks was analyzed after 24 and 48 h after serum depletion by ESI LC-MS/MS. Respective bile acid amount secreted by HepaRG^VC^ cells at 48 h was set to 1 **(A)**: Sum of all bile acids measured: cholic acid, chenodeoxycholic acid, taurocholic acid (TCA), glycocholic acid (GCA), taurochenodeoxycholic acid (TCDCA), glycochenodeoxycholic acid (GCDCA) **(B)**: Secretion of TCA and GCA **(C)**: Secretion of TCDCA and GCDCA. Shown are means and SD of 3-7 independent measurements. Statistical significance was assessed by two-way ANOVA with Bonferroni correction (*p < 0.05, **p < 0.01, ***p < 0.001, ****p < 0.0001).

Essential steps of bile acid synthesis strongly depend on POR, particularly via the microsomal activities of CYPs 7A1, 7B1 and 8B1. As we observed a strong decrease of CYP7A1 and CYP8B1 mRNA expression, we expected changes in bile acid secretion. Indeed, total bile acid secretion into the culture medium was slightly decreased in both types of HepaRG^−POR^ cells compared to HepaRG^VC^ (Figure 3A). Detailed analysis of the individual bile acids revealed differential effects on the individual species (Figures 3B,C). In agreement with other reports (Burban et al., 2019), the HepaRG cells secreted more taurine conjugated bile acids than glycine-conjugated bile acids with a ratio of taurine conjugates to glycine conjugates between 2:1 and 4:1. The secretion of both cholic acid conjugates was significantly decreased by up to 70% for taurocholic acid (TCA) and by up to 30% for glycocholic acid (GCA) in both HepaRG^−POR^ cells (Figure 3B). The conjugates of chenodeoxycholic acid showed contrary effects. Glycochenodeoxycholic acid (GCDCA) increased significantly up to 170% whereas taurochenodeoxycholic acid (TCDCA) was marginally affected (Figure 3C).

Liver specific *Por* knockout in mice led to lipid accumulation in their hepatocytes (Gu et al., 2003; Henderson et al., 2003; Porter et al., 2011), an effect reproduced in rat hepatoma cells treated with siRNA targeting Por (Porter et al., 2011). We observed accumulation of lipid droplets in both types of HepaRG^−POR^ and HepaRG^VC^ cells upon oleate treatment but found no difference between HepaRG^−POR^ and HepaRG^VC^ cells (Supplementary Figure S2).

### Effects of POR Knockdown on Protein Expression

To study the systemic effect of the POR knockout in HepaRG cells on global protein expression levels we used a label-free quantitative proteomics approach on three growth replicas per cell line each analyzed by LC-MS/MS in duplicates (n = 18). Data quality of the LC-MS analyses was ascertained by correlation analysis of protein abundances across all runs (Supplementary Figure S3). The Pearson r-value > 0.998 for duplicate measurements and higher than 0.9 for the biological replicates demonstrates the high reproducibility of the relative label-free quantification. This could be confirmed by principal components analysis (PCA, Supplementary Figure S4).

**FIGURE 4:**
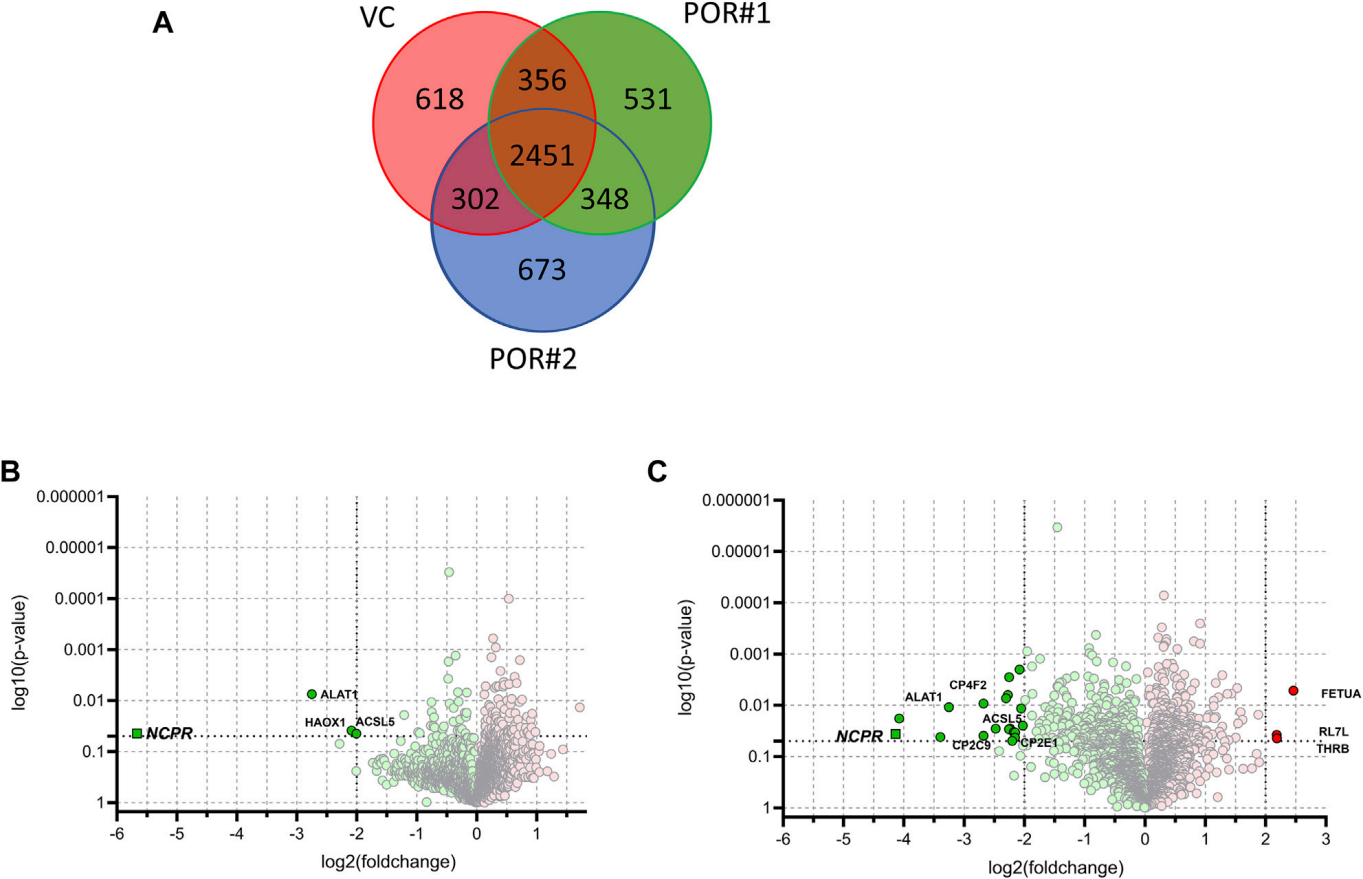
Results of label-free quantitative proteomics of HepaRG cells transduced with sgRNAs POR#1 and POR#2 and vector control (VC) (A): Venn diagram showing the unique and common proteins identified in HepaRG^VC^, HepaRG^−POR^ cells. Volcano plots representing statistical comparison of HepaRG^−POR#1^ (B) and HepaRG^−POR#2^ (C) cells to HepaRG^VC^. Two-groups *t*-test *p*-values are given on the *y*-axis with the corresponding log2 (fold changes) of protein intensities on the *x*-axis, respectively (green: lower expressed, red: higher expressed). Statistically significant proteins with *p* ≤ 0.05 and log2 (fold change) ≥2 are highlighted and selected proteins are labelled with protein names.

We identified a total of 5,279 proteins of which 2,451 were detected in at least one LC-MS injection sample of each of the three HepaRG strains. 618 proteins were uniquely identified in HepaRG^VC^, and 531 and 673 proteins were only identified in HepaRG^−POR#1^ and HepaRG^−POR#2^, respectively (Figure 4A). Significant differences between the two types of HepaRG^−POR^ cells and HepaRG^VC^ were determined by pairwise comparison of relative protein abundances using a log2 (fold change) cut-off of ≥2 and a significance level of *p* < 0.05 (*t*-test). Overall, expression differences were significant for 4 and 23 proteins in HepaRG^−POR#1^ and HepaRG^−POR#2^ cells, respectively, with the majority of proteins showing downregulation upon POR knockdown (Figures 4B,C). As expected, POR (NCPR) showed the strongest reduction in both knockdown cell lines, by 51-fold for HepaRG^−POR#1^ and 18-fold for HepaRG^−POR#2^ (Supplementary Table 1). Significantly lower protein levels were also observed for CYP2C9 (CP2C9) in both HepaRG^−POR^ strains, which is in concordance with the previous study in terms of observed immunoquantified protein expression levels and activity (Heintze et al., 2021). Other proteins showing a significant downregulation in the HepaRG^−POR^ cells include further enzymes involved in drug metabolism like CYP2E1 (CP2E1), UDP-glucuronosyltransferase 2A3 (UD2A3), and the bile salt sulfotransferase SULT2A1 (ST2A1), as well as components of lipid metabolism and homeostasis like ACSL5, ACS2B (ACSM2B), CYP4F2 (CP4F2), FABP1 (FABPL), PLIN2. Only three proteins were found with significantly higher expression in HepaRG^−POR#2^ cells, fetuin-A AHSG (FETUA), a protein associated with obesity and insulin resistance, RL7L, a ribosomal protein involved in ribosomal biogenesis, and prothrombin F2 (THRB) involved in thrombosis and hemostasis during blood clot formation.

**FIGURE 5:**
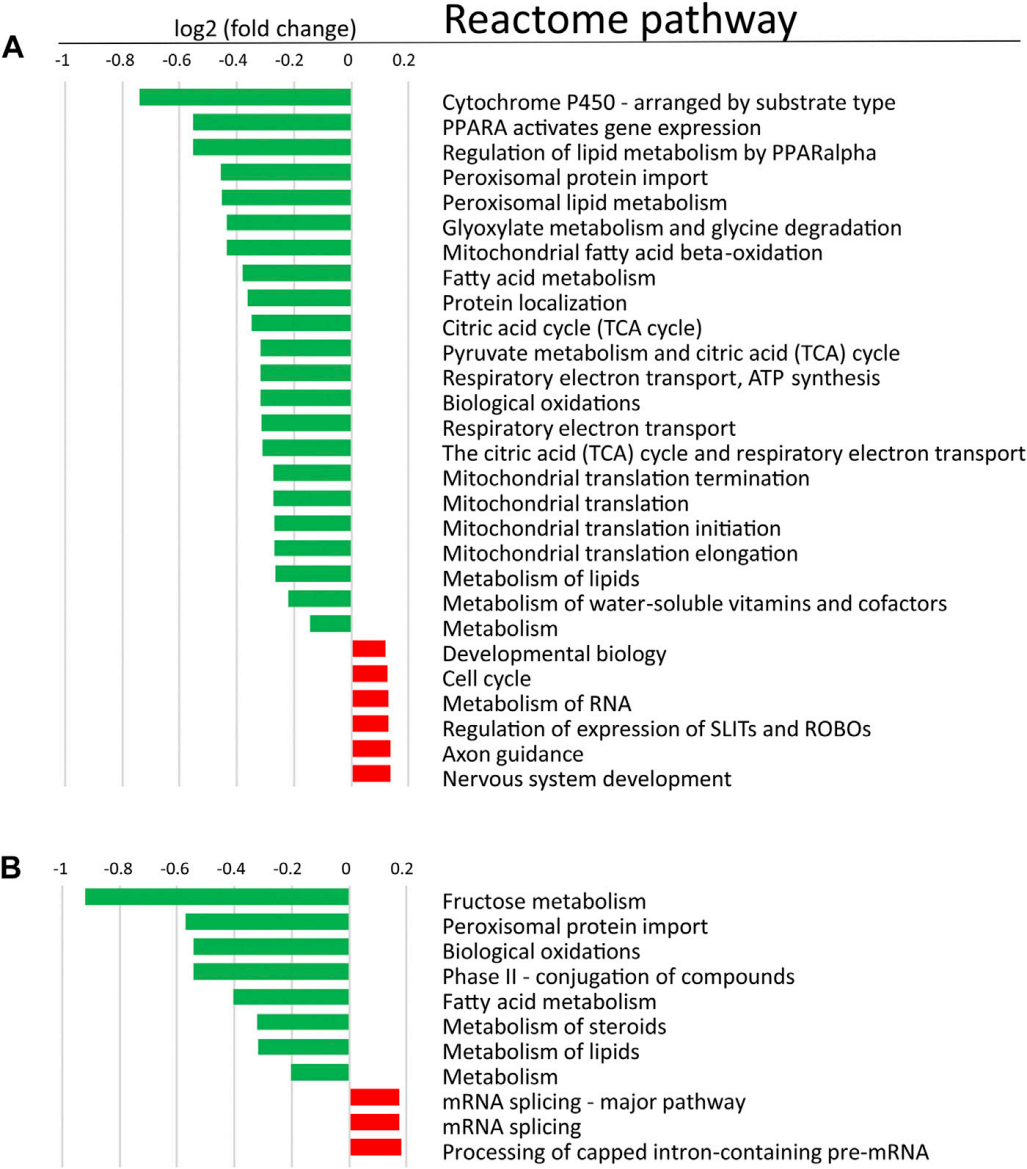
Quantitative Reactome pathway enrichment analysis. Shown are differentially enriched pathways as averaged log2 (fold change) compared to HepaRG^VC^ (FDR<0.001) performed with reactomeGSA for (A): HepaRG^−POR#1^ and (B): HepaRG^−POR#2^. green, lower expressed in knockdown-cells, red, higher expressed in knockdown cells.

In order to determine changes in biological pathways induced by POR knockdown, we used relative protein abundances between HepaRG^VC^ and the two HepaRG^−POR^ strains and performed quantitative pathway enrichment analysis using the Reactome Pathway knowledgebase for both knockdown cell lines compared to HepaRG^VC^ cells. From 2,075 quantified proteins occurring in all cell lines, 1,820 were recognized by Reactome and mapped to pathways. Compared to HepaRG^VC^, 22 and eight pathways were significantly (FDR *p* < 0.001) downregulated in HepaRG^−POR#1^ and HepaRG^−POR#2^, respectively (Figures 5A,B). Consistently, in both knockdown cell lines downregulated proteins were overrepresented in pathways of metabolism including fatty acid metabolism, metabolism of lipids, biological oxidations and mitochondrial import and translation (Figure 5, Supplementary Figures S5A,B blue).

**FIGURE 6:**
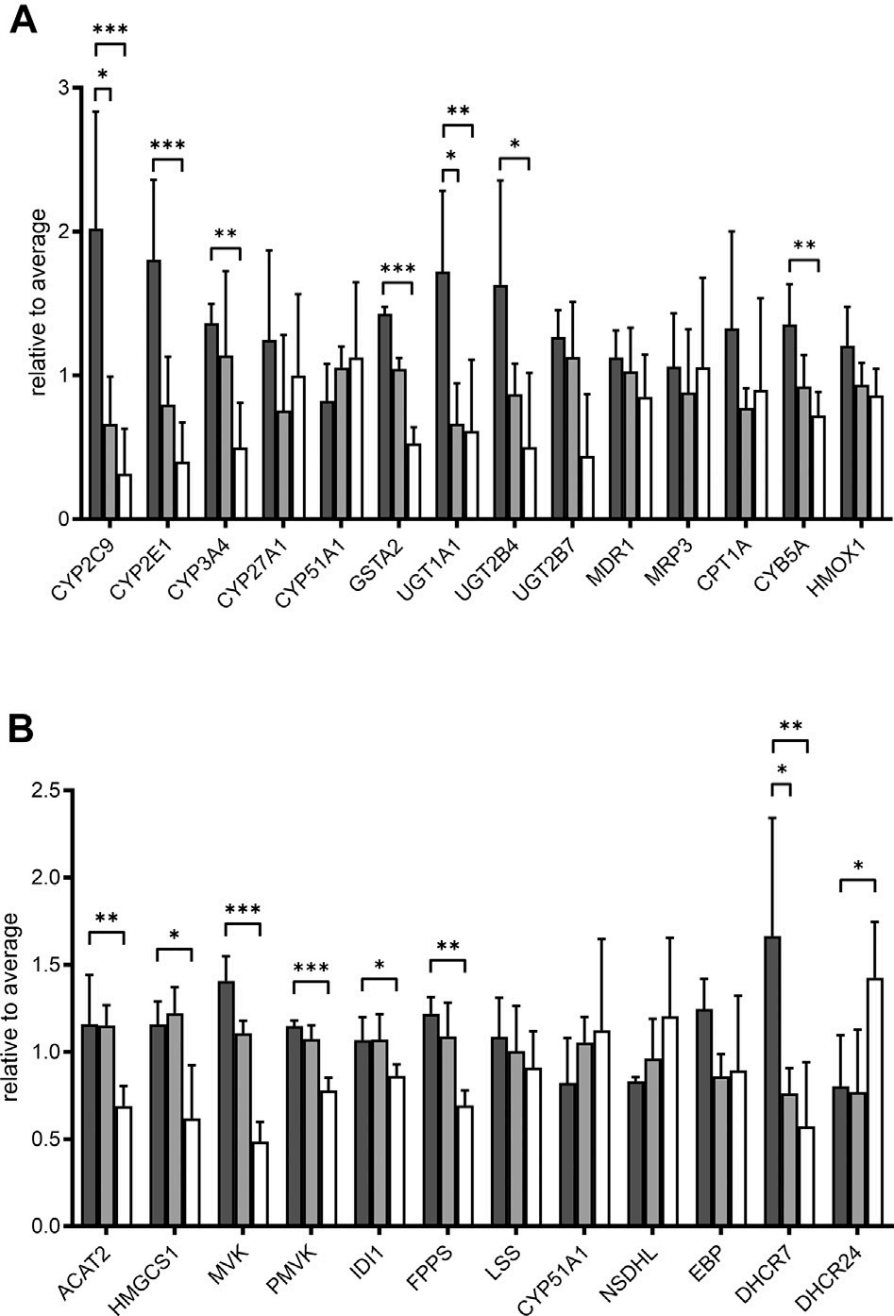
Effect of POR knockdown on selected proteins in HepaRG cells. Relative label-free quantification abundances in HepaRG cells transduced with vector control (VC, black) or sgRNAs POR#1 (light grey) and POR#2 (white) and differentiated for 2 weeks (A): Proteins corresponding to mRNA expression panel (Figure 1) (B): Enzymes in cholesterol biosynthesis. Data are normalized to average of all samples. *p < 0.05, **p < 0.01, ***p < 0.001, Kruskal-Wallis test with Dunn’s post-hoc test.

We further looked specifically at selected proteins related to POR. Proteins corresponding to genes in the expression analysis described above are shown in Figure 6A. The influence of POR knockdown on protein abundance of CYP enzymes was similar to the effect on mRNA expression. Most CYPs found were reduced upon POR knockdown with a stronger effect in HepaRG^−POR#2^ cells. In addition to CYP2C9, which was significantly reduced three fold (*p* < 0.05) and 6.4 fold (*p* < 0.001) in both HepaRG^−POR^ cells, CYP2E1 and CYP3A4 showed significant reductions in HepaRG^−POR#2^ cells (*p* = 0.033 and *p* = 0.019, respectively). Furthermore, several CYPs involved in the oxidation of PUFAs, hydroxylated fatty acids or long-chain fatty acids and requiring NADH via POR as an electron donor were significantly reduced in HepaRG^−POR#2^ cells, including CYP4F2 (CP4F2, *p* < 0.01) and CYP4F11 (CP4FB, *p* < 0.05) (Figure 4C, Supplementary Table S1). In addition to UGT2A3 (UD2A3) and SULT2A1 (ST2A1), other phase II enzymes were also downregulated upon POR knockdown including the glucuronosyltransferases UGT1A1, UGT1A6, UGT1A9, and UGT2B4 (*p* < 0.05; Supplementary Table S1). In agreement with the effect on gene expression, CYB5A was significantly decreased in HepaRG^−POR#2^ (*p* = 0.029) while HMOX1 expression was only slightly changed (Figure 6A). Due to their low abundances, transcriptional regulators e.g. of CYP enzymes like NR1I2 (PXR) and NR1I3 (CAR) could not be detected.

It has been demonstrated that POR knockdown has a strong influence on cholesterol biosynthesis and metabolism. Of 20 enzymes involved in cholesterol biosynthesis, twelve were detected in each of the three types of HepaRG cells. Proteins catalyzing the first steps of cholesterol biosynthesis to squalene, from acetyl-CoA acetyltransferase ACAT2 (THIC) to farnesyl diphosphate synthase FDPS (FPPS), were significantly reduced in HepaRG^−POR#2^ cells compared to HepaRG^VC^ while for HepaRG^−POR#1^ cells no significant reduction was observed (Figure 6B). Enzymes catalyzing the steps downstream of squalene showed varying effects. Protein expression of CYP51, which requires POR for activity, showed an >1.3 fold increase in both HepaRG^−POR^ cells, which was however not significant. The most pronounced effect with a two- or 3-fold reduction for HepaRG^−POR#1^ or HepaRG^−POR#2^, respectively, was seen for 7-dehydrocholesterol reductase (DHCR7), the terminal enzyme in cholesterol biosynthesis. Interestingly, the alternative enzyme DHCR24 (DHC24) was increased in HepaRG^−POR#2^. Of the enzymes converting cholesterol into bile acids we only detected CYP27A1 (CP27A1), which initiates the alternate bile acid synthetic pathway via 27-hydroxycholesterol and the side chain oxidation steps. Like mRNA expression, protein expression of this enzyme showed only minor changes upon POR knockdown (<1.7 fold, Figure 6A).

**FIGURE 7:**
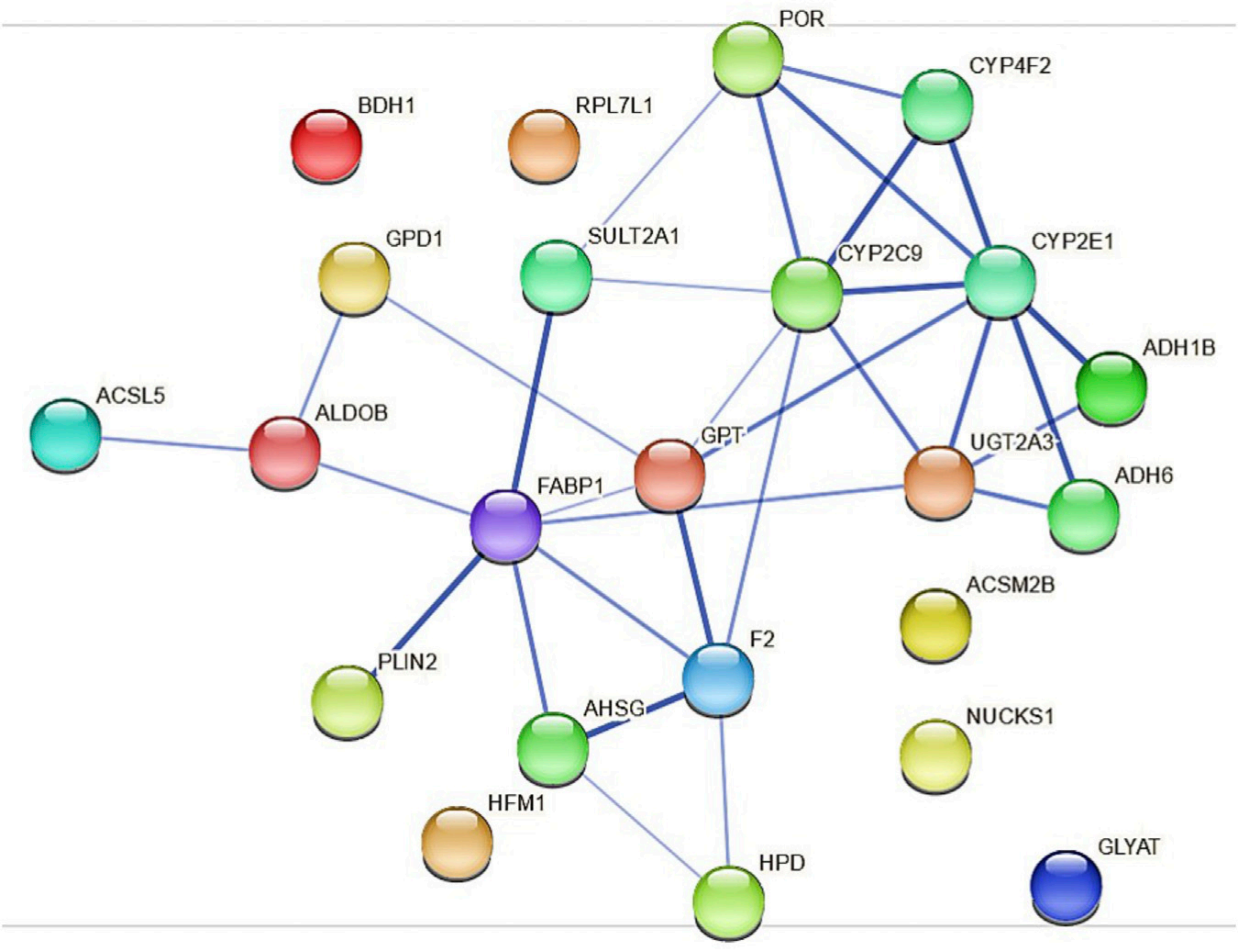
Protein-protein interactions for the 23 differentially expressed proteins identified in HepaRG^−POR#2^ cells analyzed with STRING using a minimum interaction score of 0.4 (medium confidence). Each node represents a protein and the line thickness of the edges indicates the confidence of the functional associations.

Because the protein expression differences are more pronounced in HepaRG^−POR#2^ cells we used STRING to further analyze and get more insight into possible functional links of POR knockdown to metabolic processes (Figure 7). As expected, the network map depicts the CYP enzymes CYP2C9, CYP2E1, CYP4F2 as direct interaction partners of POR with other drug metabolism related proteins like UGT2A3 and the alcohol dehydrogenases ADH1B and ADH6 as second next interaction partners. Proteins involved in lipid metabolism and homeostasis are shown as more distant and indirect interaction partners of POR. Several as confident classified interactions exist between the drug metabolism related proteins and metabolic processes, for example between UGT2A3 or SULT2A1 and FABP1 or between CYP2E1 and GPT.

## Discussion

The influence of Por on hepatic drug and endogenous metabolism has been extensively studied in mouse knockout models and recent publications addressed effects of *Por* knockout even on metabolome and proteome (Gray et al., 2020; Gethings et al., 2021). However, studies in human liver models are still missing. In our previous study we established two different CRISPR/Cas9 induced genetic *POR* knockout cell lines in the human liver cell model HepaRG, which revealed diminished CYP activities as well as a changed drug metabolizing CYP expression pattern (Heintze et al., 2021). The aim of the present study was an in-depth analysis of effects of deminished POR levels in the HepaRG cells with focus on genes involved in the absorption, distribution, metabolism, and excretion (ADME) and related regulators. We found consistent decrease of mRNA expression of several regulators of hepatic CYP expression, bile acid and lipid homeostasis in both HepaRG^−POR^ cell lines, indicating that the observed downregulation of many CYP enzymes may thus be an indirect effect of diminished POR (Figure 1).

The transcriptional receptors downregulated in HepaRG^−POR^ cells (Figure 1) belong to the *NR1H* (LXR, FXR) and *NR1I* (PXR, CAR) subfamilies, both of which are involved in regulation of bile acid metabolism by sensing the metabolic environment (Endo-Umeda and Makishima, 2019). Expression of other regulators involved in sterol and lipid homeostasis, namely PPARA, NR5A2 (LRH-1) and SREBF1, were also decreased. The main axis of bile acid regulation consists of NR1H2 (FXR), NR0B2 (SHP) and NR5A2 (LRH-1), which repress the expression of the rate limiting enzyme for bile acid synthesis, CYP7A1 and, in cooperation with HNF4α, CYP8B1 (Shin and Wang, 2019). As both NR1H2 (FXR) and NR5A2 (LRH-1) are downregulated in HepaRG^−POR^ cells, a severe deregulation of bile acid homeostasis was conceivable. While NR1H2 (FXR) downregulation was also seen in liver specific *Por* knockout mice (Wang et al., 2005), FXR target gene expression (*CYP8B1*, *7A1*) was decreased in HepaRG^−POR^ cells suggesting FXR activation rather than inactivation. CYP7A1 and 8B1 as FXR targets were among the four strongest decreased phase I enzymes measured in HepaRG^−POR^, while expression of CYP27A1, involved in the alternative bile acid synthesis pathway, was not changed. In addition, deregulation of lipid and cholesterol biosynthesis is indicated due to the decreased mRNA expression of other enzymes involved in these metabolic processes (CYP4A11, CYP51, SCD, CYB5A, FABP1), which are target genes for PPARα, LXRα and SREBF1 (Debeljak et al., 2000; Antoun et al., 2006; Paton and Ntambi, 2009; Guzmán et al., 2013). By contrast, AHR/ARNT as transcriptional components of the AhR (aryl hydrocarbon receptor) pathway, which regulate CYP1A1 and 1A2 in particular, were not changed significantly. Therefore, induction of the AhR pathway by endogenous ligands such as arachidonic acid derivatives (Larigot et al., 2018) or chenodeoxycholic acid (CDCA) (Ibrahim, 2015) may lead to a compensatory increase of CYP1A expression.

In liver specific *Por* knockdown mice gene expression phenotypes are ascribed to activation of CAR, SREBP, PPARγ and Nrf1 and repression of PPARα and FXR (Wang et al., 2005; Weng et al., 2005; Cheng et al., 2014a). This results in an induction of alternative detoxification enzymes in liver and the small intestine, which appears to partially compensate for the loss of microsomal P450 function. In addition, Nr1h4 (FXR) inhibition leads to increased Cyp7a1 and 8b1, two major CYP enzymes involved in bile acid synthesis, but decrease of Nr0b2 (SHP) mRNA expression. Alterations in PPARα target gene expression may be causative to a decrease in fatty acid oxidation but an increase in fatty acid uptake, which subsequently accounts for the severe hepatic lipidosis observed in mice and cell culture experiments (Gu et al., 2003; Henderson et al., 2003; Weng et al., 2005; Porter et al., 2011). However, we did not observe a comparable response of lipid accumulation in our model (Supplementary Figure S2), which may be explained not only by the apparent FXR inactivation instead of activation (Porter et al., 2011) but also by more pronounced effects on lipid droplet formation in the rodent system (Li and Chiang, 2014).

*De novo* cholesterol biosynthesis is POR dependent due to the enzymatic activity of the lanosterol-14α-demethylase (CYP51). POR knockdown in HepaRG cells lead to the accumulation of lanosterol while the cholesterol precursor lathosterol was unchanged (Figure 2). This finding is in accordance with observations in hepatic *Por*-null mice or in rat hepatoma cells with suppression of Por expression by siRNA (Porter et al., 2011; Porter, 2012).

In humans, POR influences bile-acid homeostasis via CYP7A1, 7B1, 8B1, and indirectly impacts FXR and PXR signaling via bile acids as endogenous ligands (Pols et al., 2011; Chiang and Ferrell, 2018). CDCA, TCA, TCDCA are known agonists of FXR, while GCDCA and other more hydrophilic derivates are more antagonistic factors (Juřica et al., 2016; Chiang and Ferrell, 2018). In contrast to humans, mice have an additional primary bile acid form, muricholic acid (MCA), a product of the microsomal Cyp2a70 (Takahashi et al., 2016). MCA and taurine conjugates of MCA are no agonists or even antagonists of FXR (Sayin et al., 2013; Li and Dawson, 2019) which underlines that bile acid regulation varies majorly between different species. Taking the mRNA expression data as a basis, changes in the bile acid composition of HepaRG^−POR^ cells is suggested. While the whole bile acid pool in HepaRG^−POR^ cells was only slightly decreased, individual bile acid species were highly differentially affected by POR knockdown (Figure 3). Slightly increased CDCA conjugates in HepaRG^−POR^ cells may indicate increased bile acid synthesis via the alternative acidic pathway with the POR-independent, mitochondrial CYP27A1 (Monte et al., 2009) predominantly leading to CDCA synthesis (Alnouti, 2009; Pikuleva and Waterman, 2013). Enhanced alternative bile acid synthesis was also reported in *Por* knockout mice, where an increase in CDCA and TCDCA in the liver was observed (Cheng et al., 2014b). The observed downregulation of nuclear receptor expression in our cell model could be in response to the altered bile acid composition with decreased levels of TCA and GCA and unchanged TCDCA levels while levels of GCDCA were increased. Under physiological healthy conditions, FXR is usually not activated. Higher levels of CDCA lead to FXR activation (Juřica et al., 2016; Chiang and Ferrell, 2018), which may explain decreased CYP7A1 and 8B1 mRNA expression levels. Increased CDCA levels could also influence HNF4α target gene expression, as CDCA reduces the transactivation potential of HNF4α via induction of the expression of the co-repressor SHP, thereby downregulating for instance the expression of endogenous SLC22A7 (OAT2) mRNA in Huh7 cells (Li et al., 2019). We also found a decrease of SLC22A7 mRNA expression up to 0.46-fold lower in HepaRG^−POR#2^ (Figure 1), however not statistically significant. Furthermore, we observed a higher percentage of taurine conjugates compared to glyco-conjugates as already described by others (Sharanek et al., 2015; Burban et al., 2019). Thus, regarding bile acid composition with increased GCDCA and reduced TCA levels, HepaRG cells seem to resemble hepatocytes originating from NASH (non alcoholic steatohepatitis) or cholestatic patients (Trottier et al., 2012; Lake et al., 2013) rather than “healthy” primary human hepatocytes (Sharanek et al., 2015) due to antagonistic effect of GCDCA on FXR. In disease state bile acid signaling is known to show large species differences. Cholestasis in humans is associated with suppressed bile acid synthesis, while rodents have increased bile acid synthesis (Straniero et al., 2020). It should be noted that bile acid homeostasis also involves systemic regulation and a tight interplay between liver and intestine (Cheng et al., 2014b; Chiang, 2017). . This aspect is of course not represented by our HepaRG cell model.

As indicated by the mRNA expression analysis, POR knockdown in HepaRG cells influences gene expression on a global level (Figure 1). Consistently, the untargeted proteomics analysis demonstrated reduced expression of several drug metabolism-related proteins (Figure 6A), again contrasting findings in liver specific *Por* knockout mice (Wang et al., 2005; Weng et al., 2005; Mutch et al., 2007; Henderson et al., 2013; Gethings et al., 2021). Of particular interest are the data regarding the protein PLIN2 (perilipin-2), one of the top lower expressed (up to 10fold) proteins in HepRG^−POR^, and FETUA (AHSG, alpha 2-HS glycoprotein) the most upregulated (up to 5fold) protein with diminished POR. PLIN2 has been described to impair hepatic lipid accumulation and *Plin2* whole body gene deletion prevented obesity and insulin resistance in Western diet-fed mice by suppressing hepatic SREBP-1/2 activity (Libby et al., 2016). This may counteract lipid droplet formation upon POR depletion, thus explaining the absence of lipid accumulation in our cell system. On the other hand, FETUA (AHSG, alpha 2-HS glycoprotein), a plasma glycoprotein predominantly synthesized in the liver, is increased in type 2 diabetes mellitus, metabolic syndrome, and nonalcoholic fatty liver disorder (NAFLD) (Bourebaba and Marycz, 2019). Pathway analysis revealed severe downregulation of a series of metabolic pathways, especially fatty acid metabolism, metabolism of lipids, biological oxidations as well as phase I and phase II drug metabolism (Figure 5). This illustrates the general role of POR in endogenous and xenobiotic metabolism which is in line with previous analyses of the lipid and bile acid metabolome as well as proteomic analyses in liver specific *Por* knockout mice (Gray et al., 2020; Gethings et al., 2021). Only few proteins show higher expression levels upon POR knockdown in the HepaRG cell model. These were mapped to six and three pathways in HepaRG^−POR#1^ and HepaRG^−POR#2^, respectively, compared to HepaRG^VC^. These include metabolism of RNA, mRNA splicing, and processing of pre-mRNAs (Figures 5A,B, Supplementary Figures S5A,B yellow). Nevertheless, both knockdown cell lines are well comparable in downregulated genes and thereby affected pathways. Only effects for a small number of affected pathways are more pronounced in the HepaRG^−POR#2^model (Figure 5, Supplementary Figure S5). POR exhibits a particular role in the cholesterol biosynthesis illustrated by protein expression changes in this pathway (Figure 6B). Biosynthesis of cholesterol can occur by two separate pathways, namely the Kandutsch-Russell and Bloch pathways, with the latter being more prominent in the liver (Mitsche et al., 2015). Several enzymes in these pathways were significantly downregulated with 7-dehydrocholesterol reductase (DHCR7), the terminal enzyme in cholesterol biosynthesis in the Kandutsch-Russell pathway being the most affected. Interestingly, the linking enzyme between the two pathways, 24-dehydrocholesterol reductase (DHCR24), was increased by POR knockdown. This compensatory response was in agreement with already described in *Dhcr7* knockout mice (Koczok et al., 2019).

Using STRING analysis, we could confirm the central role of POR in drug metabolism (Figure 7). As already discussed above for the mRNA expression analysis (Figure 1), an indirect consequence of POR knockdown is the change in various hepatic signaling pathways (Supplementary Figure S6). This is depicted by the interaction of several alcohol dehydrogenases with CYP2E1, indicating an activation of the AhR/ARNT signaling pathway by AhR ligands (Attignon et al., 2017). In addition, several interactions indicating towards changed PPARα signaling were revealed (eg. FABP1 and SULT2A1, PLIN1 and ALDOB).

In conclusion, gene and protein expression changes in human hepatocytes seem to be a coordinated response to altered endogenous metabolite levels, mainly bile acids, which is generally in line with findings in liver specific *Por* knockout mice (Mutch et al., 2007; Gray et al., 2020; Gethings et al., 2021). However, species differences make it difficult to translate specific findings concerning biological mechanisms from *Por* knockout mice to the human system. Our genetic POR knockout human HepaRG cell model illustrates marked differences especially in bile acid composition and signaling to rodent animal models and may provide new insights into the importance of POR in endogenous cell metabolism.

## Data Availability

The datasets presented in this study can be found in the ProteomeXchange Consortium via the PRIDE partner repository (http://www.ebi.ac.uk/pride), accession number PXD028577.
