# Supplementary material for: Effects of Diminished NADPH:cytochrome P450 Reductase in Human Hepatocytes on Lipid and Bile Acid Homeostasis
Source: Front Pharmacol. 2021 Nov 15;12:769703. doi: 10.3389/fphar.2021.769703 (PMC8634102; doi:10.3389/fphar.2021.769703)
Supplement: Supplementary file 1 [file DataSheet1.pdf]

Supplementary Material

Supplementary Table S1: List of identified protein entities (n=3,069) with individual quantitative data retrieved from all measured samples, group comparison fold changes and and t-test p-values with Benjamini-Hochberg adjusted p-values for each HepaRG^{-POR} vs HepaRG^{VC} calculated with Genedata Analyst.

(provided as excel file)

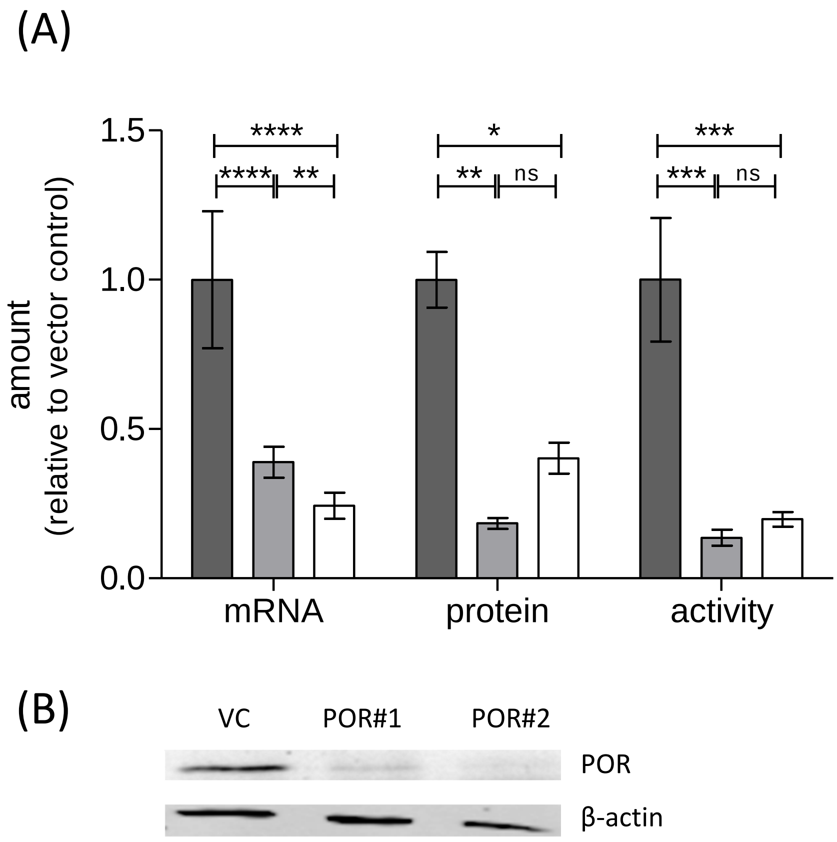

Supplementary Figure S1: POR expression in HepaRG genetic POR knockout celllines established by CRISPR/Cas9 technology. (A) POR expression and cytochrome c reductase activity in transduced differentiated HepaRG cells. POR mRNA was quantified in total RNA from cell lysates and POR protein and cytochrome c reductase activity were quantified in microsomal fractions. Mean levels ± SD are shown relative to vector control (VC) set at 1 (dark grey: VC, light grey: POR#1, white: POR#2) (graph taken from Heintze et al., Scientific reports 11:1, 1000 (2021)). (B) Example Western blot of immunostained POR protein (77kD) and ß-actin (42kD) according to Heintze et al., Scientific reports 11:1, 1000 (2021).

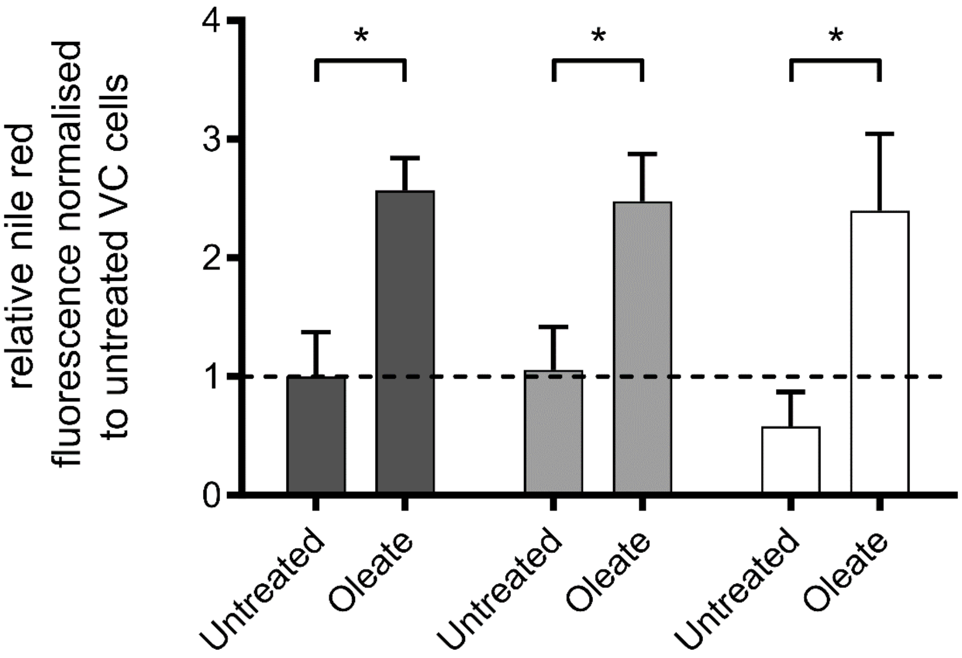

Supplementary Figure S2: Lipid droplet accumulation in HepaRG cells. For analysis of lipid droplet accumulation using Nile Red staining HepaRG cells were transduced with vector control (VC, dark grey) or sgRNAs POR#1 (light grey) and POR#2 (white), differentiated for two weeks and treated with/without 300 µM oleate for additional 48 h. Respective Nile Red fluorescence in untreated HepaRG^{VC} cells was set to 1. Shown are means and SD of three independent measurements. Statistical significance was assessed by paired t-test (*$p < 0.05$).

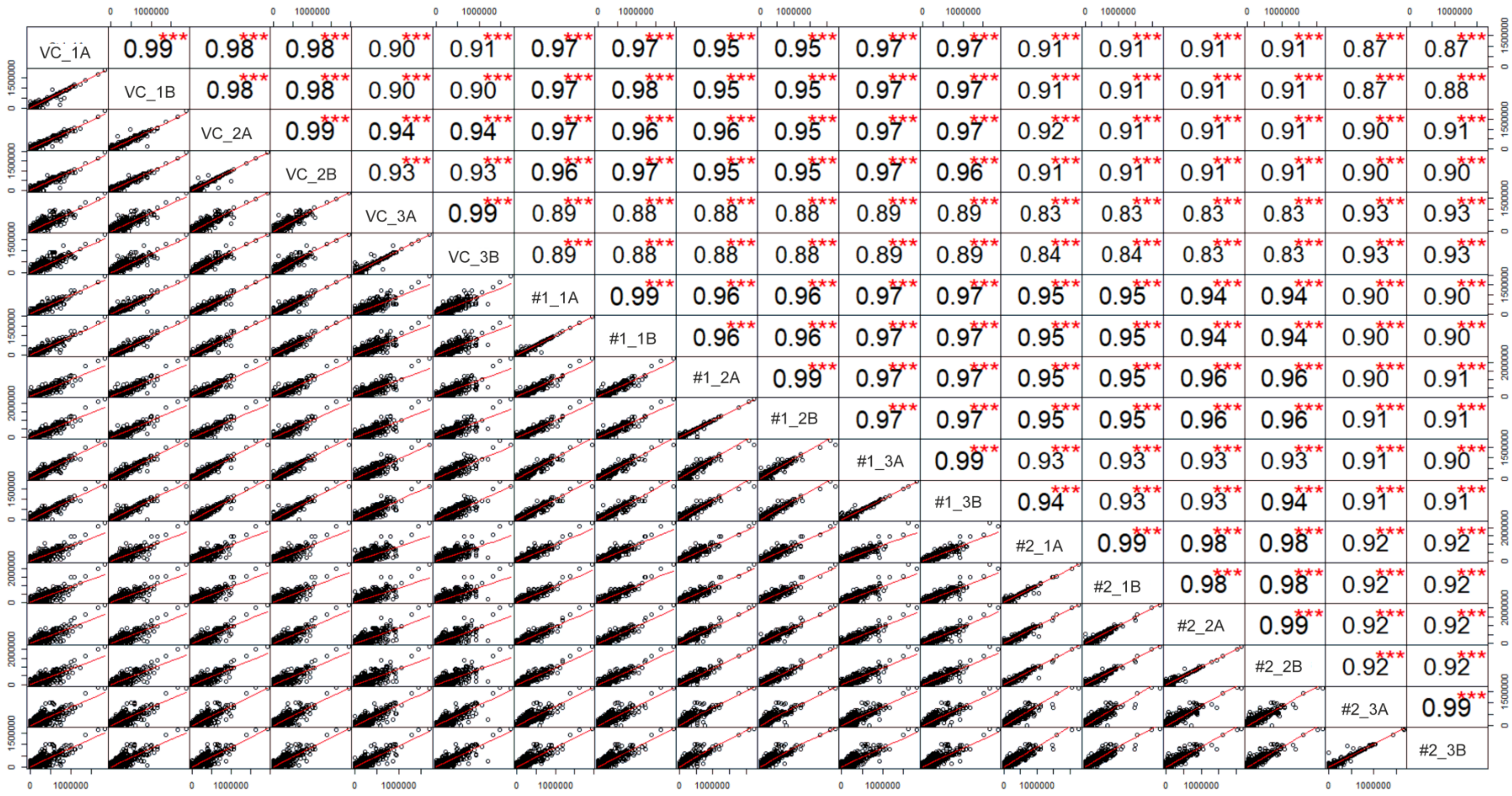

Supplementary Figure S3: Protein abundance correlation plots with Pearson's correlation score of all quantified proteins in all measured samples.

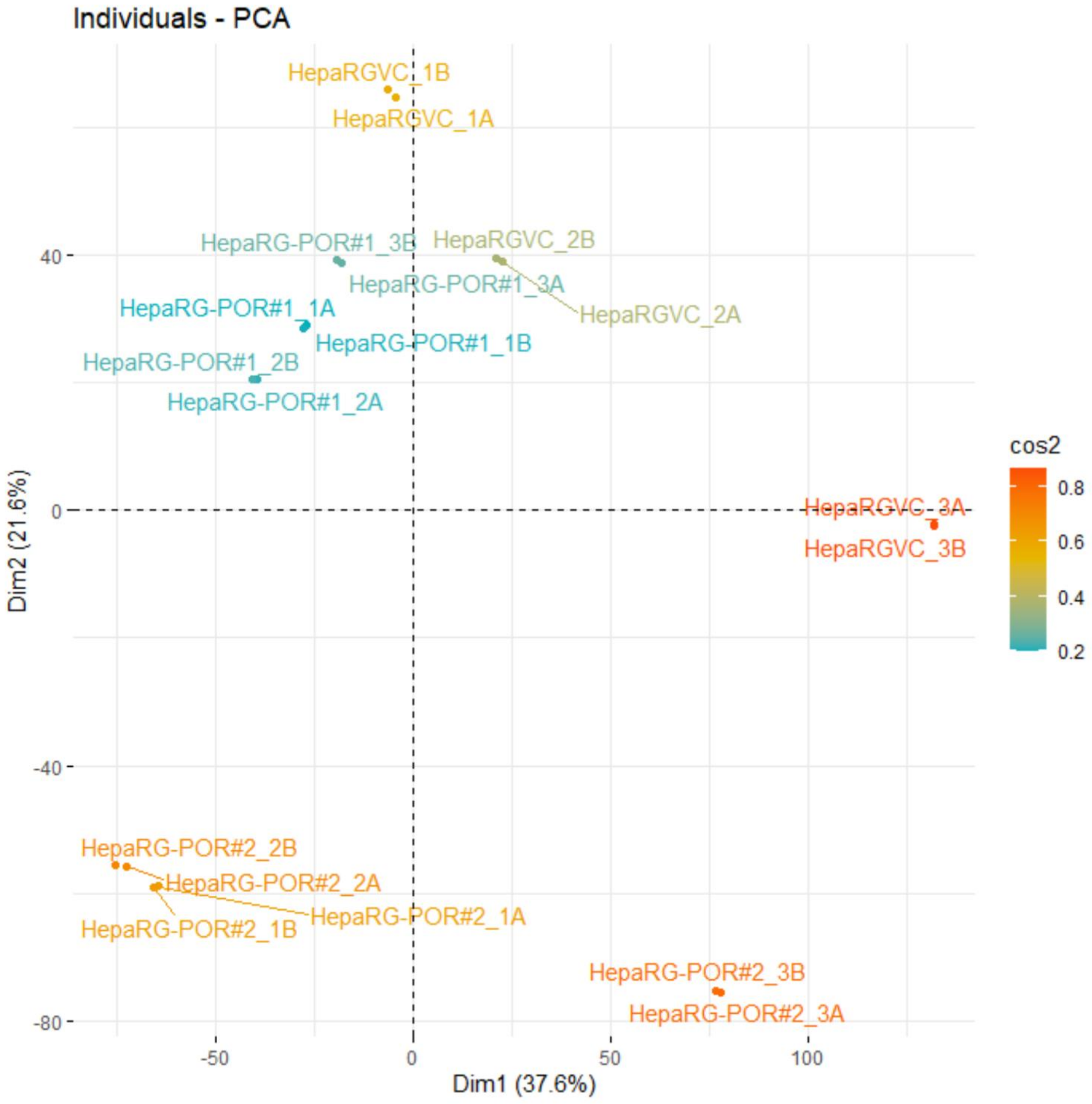

Supplementary Figure S4: Principal component analysis (PCA) score plot of label-free quantitative proteomics data. Squared cosinus quality measure (cos^2) for individual samples is shown in color according to given scale.

(A)

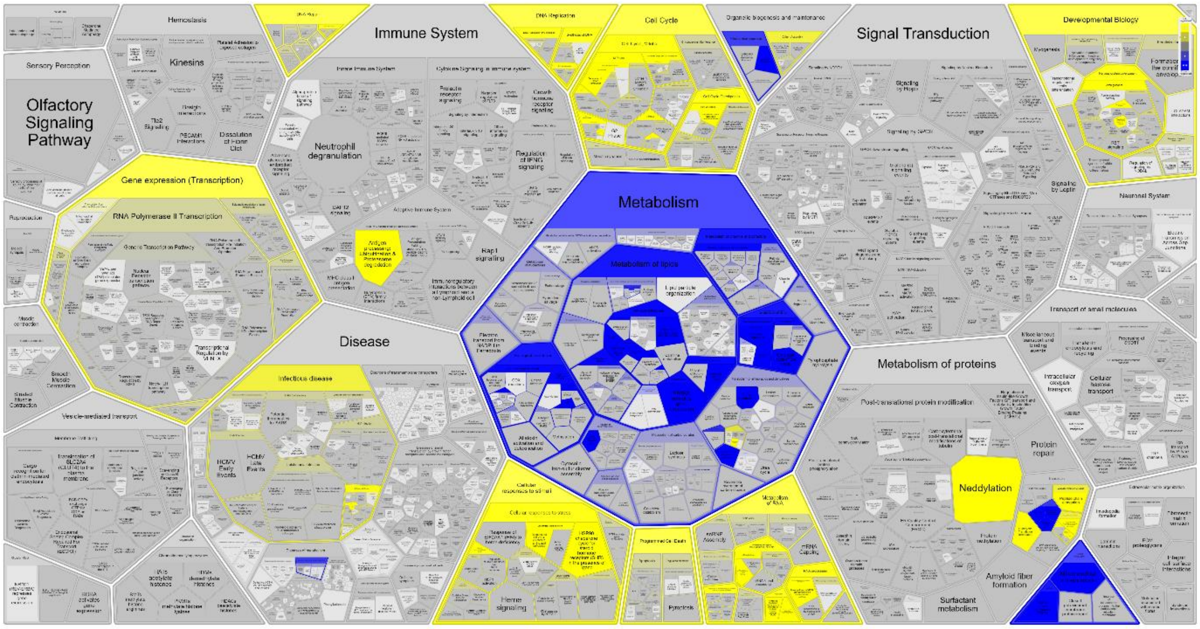

(B)

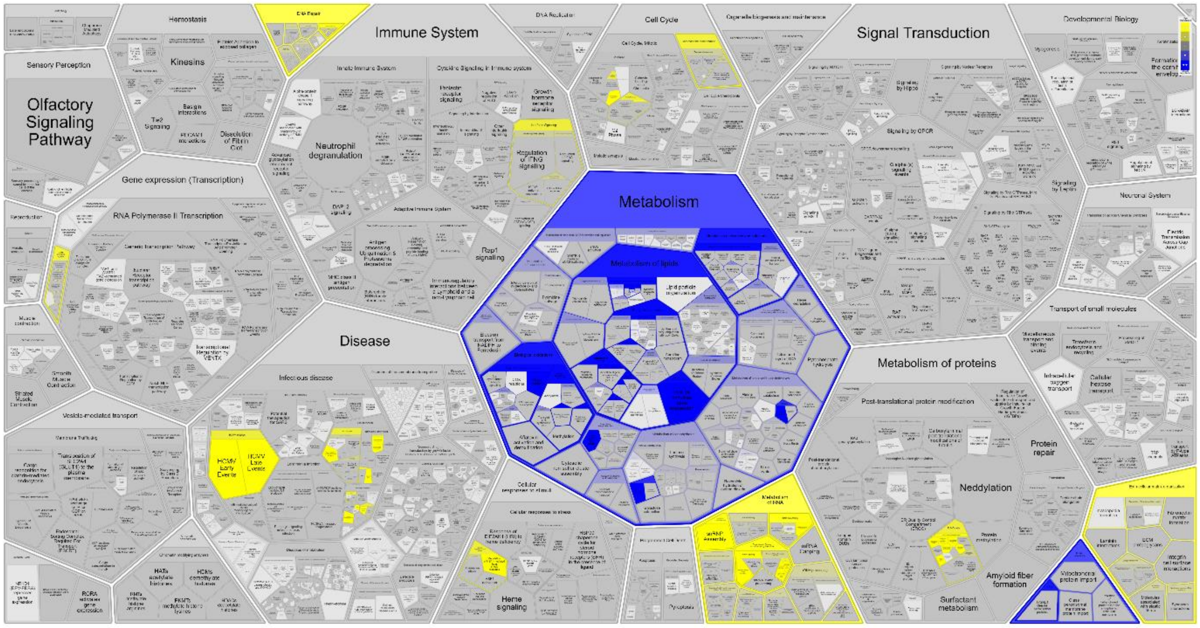

Supplementary Figure S5: Quantitative pathway analysis of proteomics data. POR knockdown cells compared to vector control (VC) cells. (A): HepaRG$^{-POR\#1}$; (B): HepaRG$^{-POR\#2}$. Expression differences of overrepresented pathways p<0.001 are illustrated as Voronoi graph in reactome.org. Blue: down regulated in knockdown cells, yellow: upregulated in knockdown cells, shaded colors are displayed for not significantly enriched pathways.

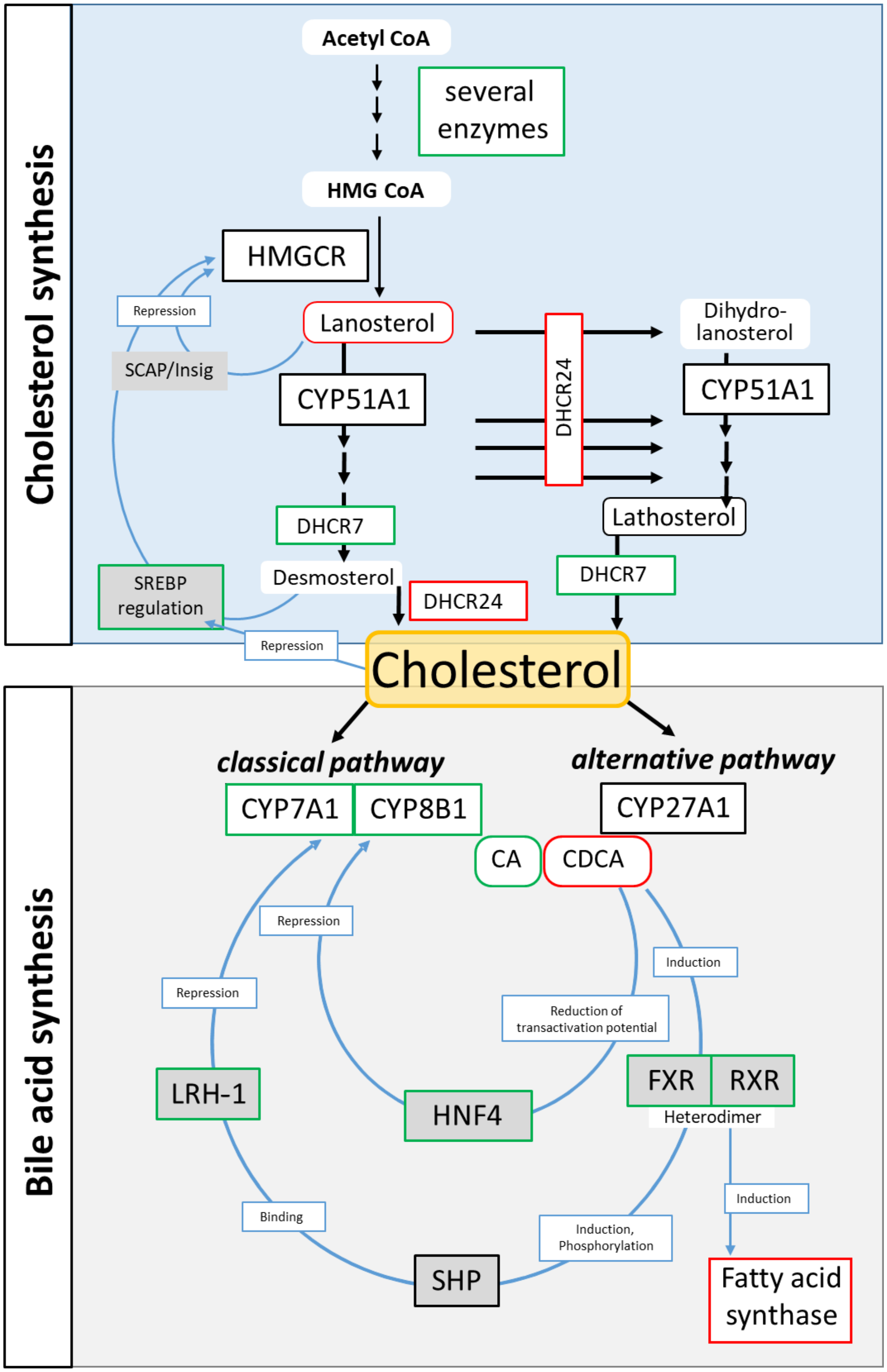

Supplementary Figure S6: Scheme of cholesterol metabolic pathways affected by diminished POR expression. Upper panel: changes in cholesterol biosynthesis; lower panel: changes in bile acid synthesis. Increased abundance of proteins or metabolites is shown in red, decreased in green, unchanged in black. Regulatory relationships are indicated by blue boxes and arrows.
